# Repeatability of keratometry depending on tear film osmolarity

**DOI:** 10.1111/aos.70117

**Published:** 2026-03-05

**Authors:** David Schneider, Klemens Paul Kaiser, Petra Davidova, Thomas Kohnen, Tyll Jandewerth

**Affiliations:** ^1^ Department of Ophthalmology Goethe‐University Frankfurt Germany

**Keywords:** dry eye disease, keratometry, tear film osmolarity, vector analysis

## Abstract

**Purpose:**

To determine the repeatability of keratometry and astigmatism measurements of patients depending on the tear film osmolarity using three different devices.

**Methods:**

In this study, 97 eyes of 97 patients with a mean age of 64.92 ± 12.07 years received three repeated measurements with the IOLMaster 700 (Carl Zeiss Meditec, Jena, Germany), Pentacam AXL (Oculus Optikgeräte GmbH, Wetzlar, Germany) and Galilei G6 (Ziemer Ophthalmic Systems, Port, Switzerland). After that, tear film osmolarity was obtained with the TearLab Osmolarity System (TearLab Corporation, San Diego, CA, USA) for quantification of dry eye disease. Four groups were formed depending on tear film osmolarity: group 1 (<300 mOsm/L), group 2 (300–310 mOsm/L), group 3 (311–320 mOsm/L) and group 4 (>320 mOsm/L). Intraclass correlation coefficient (ICC) and vector analysis were calculated to assess the repeatability of keratometry and astigmatism measurements.

**Results:**

ICC values for keratometry were 0.957 or higher in all osmolarity groups. Highest ICC values for astigmatism were in group 1 with the Pentacam (0.986) and in group 2 (0.979), group 3 (0.951) and group 4 (0.950) with the IOL‐Master 700 respectively. Highest astigmatism ICC value for the Galilei G6 was 0.884 in group 4 and the lowest in group 3 (0.405). Vector analysis of astigmatism showed no statistically significant difference between the osmolarity groups and IOL‐Master 700 (*p* = 0.947), Pentacam (*p* = 0.353) and Galilei G6 (*p* = 0.660).

**Conclusion:**

Tear film osmolarity showed no consistent association with the repeatability of keratometry or astigmatism measurements for the IOLMaster 700 and Pentacam AXL whereas the Galilei G6 demonstrated lower repeatability overall.

## 1 INTRODUCTION

Accurate assessment of corneal tomography and ocular biometry is crucial in contemporary ophthalmology, particularly for the diagnosis and monitoring of corneal ectatic diseases, surgical planning in refractive (e.g. keratorefractive surgery) and cataract procedures and postoperative follow‐up. Several advanced imaging platforms are currently available, including Scheimpflug‐based devices, swept‐source optical coherence tomography (SS‐OCT) biometers and hybrid systems that combine multiple imaging modalities. These technologies provide high‐resolution, three‐dimensional reconstructions of the anterior segment and are widely used for obtaining reliable measurements of corneal curvature, elevation and pachymetry as well as axial length and lens power calculations (Jiang et al., 2022; Jin et al., 2023; Juliane et al., 2022; Shajari et al., 2017).

The Pentacam AXL (Oculus Optikgeräte GmbH, Wetzlar, Germany), as one example of the latest generation of Scheimpflug imaging devices, integrates corneal tomography with optical biometry, thereby broadening its diagnostic application and impact on surgical planning. Similarly, the IOLMaster 700 (Carl Zeiss Meditec AG, Jena, Germany) utilizes SS‐OCT technology and reflection‐based keratometry to provide precise measurements of axial length, keratometry, anterior chamber depth and lens thickness and has become a reference standard in modern cataract surgery planning. The Galilei G6 (Ziemer Ophthalmic Systems AG, Port, Switzerland) combines dual Scheimpflug tomography with Placido disc topography and optical biometry, offering a comprehensive evaluation of corneal morphology, posterior corneal curvature and ocular dimensions in a single device (Michael et al., 2023; Moshirfar et al., 2022; Wan et al., 2022).

For all these diagnostic instruments, measurement repeatability and reliability are essential, since even minor variability may significantly influence clinical decision‐making, such as toric intraocular lens calculation or the early detection of subtle corneal changes (Kreps et al., 2020; Ramsauer et al., 2024). While the repeatability of earlier generations of these devices has been extensively studied, less is known about factors that may compromise measurement stability in real‐world settings (Lwowski et al., 2025). One critical determinant is the quality and quantity of the precorneal tear film, which constitutes the first refractive interface of the eye. Instability of the tear film or increased osmolarity, both hallmarks of dry eye disease (DED), may introduce variability in corneal tomography and optical biometry by affecting image quality and consistency (Gjerdrum et al., 2020; Guven, 2022; Hiraoka et al., 2022).

A recent study investigated the prevalence of DED and abnormal tear osmolarity among over 200 patients scheduled for cataract surgery, highlighting that more than 55% had DED according to the criteria stipulated by the Tear Film & Ocular Surface Society Dry Eye Workshop (TFOS/DEWSII®), and almost 66% had abnormal osmolarity (Graae Jensen et al., 2023). Given the high prevalence of DED and its potential impact on ocular surface regularity, understanding its influence on tomographic measurement repeatability is of clinical relevance. The present study, therefore, investigates the repeatability of corneal tomographic parameters obtained with the IOLMaster 700, the Pentacam AXL and the Galilei G6 in eyes with DED in relation to tear film osmolarity.

## 2 METHODS

### 2.1 Study design

This prospective study was performed at the Department of Ophthalmology, Goethe‐University Frankfurt, Germany, between March 2022 and October 2025. The study protocol was approved by the local ethics committee (approval number: 2021‐433) and the principles of the Declaration of Helsinki were followed. All patients provided informed consent.

### 2.2 Inclusion and exclusion criteria

All patients with age ≥18 years who had otherwise healthy eyes and who visited the cataract consultation hour at the Department of Ophthalmology were asked to participate in the study. Exclusion criteria were any prior intraocular surgery or trauma, any active ocular pathology that could affect the planned measurements like acute or chronic corneal diseases, any eye drops application 30 min before the measurements and contact lens wearing 2 weeks prior to the study visit.

### 2.3 Measurements

Three repeated measurements on one eye were performed using Pentacam AXL (Version 1.33r03, Oculus Optikgeräte, Wetzlar, Germany), Galilei G6 (Version 2.6.0, Ziemer ophthalmic systems AG, Port, Switzerland) and the IOLMaster 700 (Version 1.90.12.05, Carl Zeiss Meditec AG, Jena, Germany). Sequence of measurements with the three devices and the study eye of the patient were randomized by drawing a sequence number out of a raffle box with equal amount of each sequences in it. After each measurement, the patient was instructed to move their head away from the headrest, stand up from the chair and then sit down again to ensure independent measurements. Only measurements with good quality feedback of the devices were used for further evaluation.

Following these measurements, tear film osmolarity was measured with the TearLab Osmolarity System (TearLab Corporation, San Diego, CA, USA). After applying the test card to the test pen, the information ‘ready’ on the test station was waited up. The patient was advised to look straight. The tip of the test pen was placed above the temporal lower eyelid without pulling it down. Then, the tip of the test pen was lowered down until it touched the tear film between eyelid and eye, beeped and turned green after successful tear collection. The test pen was then placed back into the test station, and the test result was shown on the test station screen.

All measurements were performed by the same blinded study doctor.

### 2.4 Statistical analysis

Descriptive data were presented as mean ± standard deviation (SD) and range, or as median (interquartile range; IQR) depending on the distribution of the data. Normal distribution was analysed using the Shapiro–Wilk test. Unifactorial analysis of variance was used if the data were not significantly different from a normal distribution. Otherwise, the Friedman test or the Kruskal–Wallis test was applied. For pairwise analysis, the Wilcoxon rank‐sum test was used. The data were Bonferroni corrected where necessary. The statistical significance level was *p* = 0.05. For statistical analysis, SPSS (Version 29.0.1.0, IBM Corporation) and Excel (Version 16.100.2, Microsoft Corporation) were used.

The sample size calculation relies on the following assumptions: a significance level of alpha = 0.0125 and a test strength of 80%, a clinically relevant mean difference of 0.5 and a standard deviation of 0.43 dioptre for the keratometry readings.

Then, a case number of at least 22 patients per group was needed to achieve a test power of 80%. To compensate for a drop‐out rate of about 5%, data from at least 23 patients per group and a total of 92 patients should be included in the study.

### 2.5 Data analysis

The patient collective was divided into four groups depending on tear film osmolarity: group 1 ≤ 299 mOsm/L, group 2 300–309 mOsm/L, group 3 310–319 mOsm/L and group 4 ≥ 320 mOsm/L. This classification is based on the grading system of a modified DED severity scale by Sullivan et al. (2010).

The magnitude of corneal astigmatism is calculated by the subtraction of the flat keratometry values (*K*_1_) from the steep keratometry values (*K*_2_) with the meridian corresponding to *K*_2_. The results were then converted to rectangular vectors J0 and J45 using the following equations:

$$J0=-\left(K_{2}-K_{1}\right)/2\times \text{cos2}\alpha$$

$$J45=-\left(K_{2}-K_{1}\right)/2\times \text{sin2}\alpha$$

where (*K*_2_ – *K*_1_) corresponds to the positive cylindrical power, while α is the axis corresponding to *K*_2_. Vector magnitude was then calculated with the following equation: $\sqrt{J0^{2}+J45^{2}}$.

### 2.6 Repeatability

To determine the repeatability of the three devices used for the three consecutive measurements, within‐subject standard deviation (SWSD) and intraclass correlation coefficient (ICC) values with 95% confidence intervals were calculated. ICC values were calculated using a two‐way mixed‐effects model (person = random, measurement = fixed) with absolute agreement and single‐measure reliability.

SWSD values were assessed by calculating the mean values and it's standard deviation obtained from the repeated measurements. To calculate the ICC, the ratio of between‐subject variance to the sum of the combined between‐subject and within‐subject variance was used. Correlation analysis of Δ*Ast* and SWSD was performed using the Pearson correlation coefficient.

Vector analysis was performed to assess astigmatism repeatability by computing the arithmetic mean value of vector differences among the three repeated measurements with the following equation introduced by Wang et al. (2024):

$$\Delta \mathrm{Ast}=\frac{\left|\vec{\mathrm{Ast}1}-\vec{\mathrm{Ast}2}\right|+\left|\vec{\mathrm{Ast}1}-\vec{\mathrm{Ast}3}\right|+\left|\vec{\mathrm{Ast}2}-\vec{\mathrm{Ast}3}\right|\;}{3}$$

where *Ast* means the measured corneal astigmatism (magnitude at meridian) and the three repeated measurements of corneal astigmatism with *Ast*1, *Ast*2 and *Ast*3, respectively. The Δ*Ast* represents the repeatability of the three corneal astigmatism measurements, and the closer Δ*Ast* is to zero, the better is the measurement repeatability.

### 2.7 Agreement

Agreement for the performed measurements was assessed by vector analysis and Bland–Altman plots. The 95% limits of agreement (LOAs) values were calculated using the mean difference ±1.96 SD of the differences between the three repeated corneal astigmatism measurements. The arithmetic mean values from each device were used for agreement analysis.

In the Bland–Altman plots, the *y*‐axis represents the mean difference among the three astigmatism measurements, while the *x*‐axis represents the mean of the three individual astigmatism measurements.

## 3 RESULTS

The study included 97 eyes of 97 patients (52 female [53.61%]). Mean age was 64.9 ± 12.1 years (range 25.9–90.2 years). All other demographic data including the different osmolarity groups are shown in Table 1.

**TABLE 1:** Patients' demographic and characteristics of all four groups.

Characteristics	Group 1 (*n* = 23)	Group 2 (*n* = 24)	Group 3 (*n* = 24)	Group 4 (*n* = 26)	*p*‐Value^a^
Age (years; mean ± SD, range)	68.65 ± 9.61 (50–86)	66.29 ± 12.18 (37–90)	64.08 ± 11.52 (34–89)	61.04 ± 13.67 (26–82)	0.231
Sex (female; *n*; %)	14; 60.9%	9; 37.5%	11; 45.8%	18; 69.2%	0.109
Eye (right; *n*; %)	10; 43.5%	10; 41.7%	10; 41.7%	13; 50%	0.885

^a^ Kruskal–Wallis test, *p* < .05 statistically significant.

### 3.1 Repeatability

The SWSD is shown in Table 2. It showed an overall good SWSD for repeatability of all three devices. No statistically significant differences were found between the four groups and between the three devices. Pearson correlation coefficient showed no statistically significant correlation between tear film osmolarity and SWSD.

**TABLE 2:** Within‐subject standard deviation of all four groups.

Characteristics	All (*n* = 97)	Group 1 (*n* = 23)	Group 2 (*n* = 24)	Group 3 (*n* = 24)	Group 4 (*n* = 26)	*p*‐Value^a^
*IOL‐Master 700*						
K1	0.117	0.121	0.083	0.094	0.159	0.909
K2	0.068	0.060	0.060	0.060	0.078	0.979
*K*_m_	0.074	0.082	0.062	0.063	0.085	0.497
Ast*	0.126	0.097	0.082	0.103	0.157	0.730
cCT	1.906	1.460	2.342	1.612	2.072	0.665
*Pentacam AXL*						
K1	0.130	0.066	0.094	0.165	0.159	0.418
K2	0.137	0.076	0.096	0.140	0.187	0.211
*K*_m_	0.104	0.059	0.071	0.127	0.127	0.113
Ast*	0.143	0.073	0.087	0.147	0.210	0.593
cCT	2.990	2.246	2.167	3.020	4.098	0.655
*Galilei G6*						
K1	0.375	0.474	0.226	0.515	0.213	0.488
K2	0.360	0.152	0.538	0.453	0.090	0.922
*K*_m_	0.266	0.293	0.271	0.351	0.113	0.429
Ast*	0.481	0.393	0.593	0.625	0.232	0.899
cCT	2.801	2.494	2.899	3.796	1.736	0.382

Abbreviations: Ast*, astigmatism values; cCT, central corneal thickness; K1, keratometry of the flat meridian; K2, keratometry of the steep meridian; *K*_m_, mean keratometry.

^a^ ANOVA, statistically significant *p* < .05.

The ICC with the 95% confidence interval (CI) for the three devices depending on the different osmolarity groups are shown in Table 3. For the IOLMaster 700, the astigmatism ICC values were found to be good in all osmolarity groups with values higher than 0.950 (astigmatism value in group 4). For the Pentacam AXL, ICC values were good except for the astigmatism values in group 3 (0.890). For the Galilei G6, ICC for astigmatism values was found to be worse ranging from 0.405 in group 3 to 0.884 in group 4.

**TABLE 3:** Intraclass correlation coefficient (ICC) of all four groups with 95% confidence interval.

Characteristics	Group 1 (*n* = 23)	Group 2 (*n* = 24)	Group 3 (*n* = 24)	Group 4 (*n* = 26)
*IOLMaster 700*				
K1	0.996 (0.991–0.998)	0.998 (0.996–0.999)	0.998 (0.996–0.999)	0.995 (0.991–0.998)
K2	0.998 (0.996–0.999)	0.999 (0.997–0.999)	0.999 (0.998–1.000)	0.999 (0.997–0.999)
*K*_m_	0.998 (0.996–0.999)	0.999 (0.998–0.999)	0.999 (0.999–1.000)	0.998 (0.998–0.999)
Ast*	0.974 (0.947–0.988)	0.984 (0.968–0.992)	0.951 (0.899–0.978)	0.950 (0.904–0.976)
cCT	1.000 (0.999–1.000)	0.995 (0.991–0.998)	0.999 (0.998–1.000)	0.997 (0.995–0.999)
*Pentacam AXL*				
K1	0.998 (0.997–0.999)	0.997 (0.994–0.999)	0.996 (0.993–0.998)	0.996 (0.992–0.998)
K2	0.998 (0.997–0.999)	0.997 (0.995–0.999)	0.996 (0.993–0.998)	0.996 (0.991–0.998)
*K*_m_	0.999 (0.998–1.000)	0.998 (0.996–0.999)	0.997 (0.995–0.999)	0.997 (0.994–0.999)
Ast*	0.986 (0.972–0.994)	0.979 (0.958–0.990)	0.890 (0.782–0.949)	0.913 (0.825–0.959)
cCT	0.999 (0.998–1.000)	0.996 (0.992–0.998)	0.998 (0.995–0.999)	0.993 (0.987–0.997)
*Galilei G6*				
K1	0.957 (0.914–0.980)	0.991 (0.991–0.996)	0.977 (0.954–0.989)	0.992 (0.985–0.996)
K2	0.994 (0.988–0.997)	0.953 (0.907–0.978)	0.977 (0.954–0.989)	0.997 (0.995–0.999)
*K*_m_	0.984 (0.968–0.993)	0.986 (0.973–0.994)	0.987 (0.974–0.994)	0.997 (0.995–0.999)
Ast*	0.801 (0.606–0.909)	0.534 (0.086–0.783)	0.405 (−0.142–0.720)	0.884 (0.777–0.944)
cCT	0.999 (0.998–1.000)	0.995 (0.990–0.998)	0.998 (0.995–0.999)	0.998 (0.997–0.999)

Abbreviations: Ast*, astigmatism values; cCT, central corneal thickness; K1, keratometry of the flat meridian; K2, keratometry of the steep meridian; *K*_m_, mean keratometry.

When looking at the ICC of the entire patient population, IOLMaster 700 showed an ICC of 0.966 (CI 0.952–0.976), Pentacam an ICC of 0.956 (CI 0.938–0.969) and the Galilei G6 of 0.654 (CI 0.515–0.758).

**FIGURE 1:**
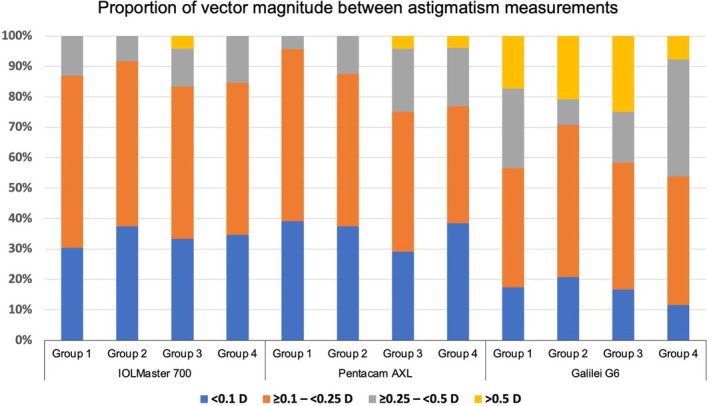
Proportion of vector magnitude for astigmatism measurements. D, Dioptres.

The proportion of vector magnitude between the three astigmatism measurements depending on osmolarity group is shown in Figure 1. There was no statistically significant difference between the four osmolarity groups (*p* > 0.571 each, Kruskal–Wallis test). While there was no statistically significant difference between IOLMaster 700 and Pentacam AXL (*p* = 0.616, Wilcoxon rank‐sum test), the Galilei G6 showed statistically significantly higher differences in vector magnitude (*p* < 0.001), respectively.

The Δ*Ast*‐values are shown in Table 4. These findings support our results of the ICC with overall worse Δ*Ast*‐values for the Galilei G6 and statistically significant differences between the devices in osmolarity groups 1 and 4.

**TABLE 4:** Repeatability of astigmatism measurements (Δ*Ast*) of all four groups (mean ± SD).

Characteristics	Group 1 (*n* = 23)	Group 2 (*n* = 24)	Group 3 (*n* = 24)	Group 4 (*n* = 26)	*p*‐Value between groups^b^
IOL‐Master 700	0.085 ± 0.049	0.076 ± 0.044	0.111 ± 0.141	0.096 ± 0.092	0.948
Pentacam AXL	0.072 ± 0.040	0.087 ± 0.075	0.129 ± 0.127	0.099 ± 0.096	0.353
Galilei G6	0.207 ± 0.206	0.215 ± 0.315	0.217 ± 0.300	0.164 ± 0.129	0.660
*p*‐Value between devices^a^	0.003	0.223	0.167	0.015	

Abbreviation: SD, standard deviation.

^a^ Friedman test.

^b^ Kruskal–Wallis test; *p* < 0.05 statistically significant.

Pearson correlation coefficient showed no statistically significant correlation between tear film osmolarity and Δ*Ast*.

### 3.2 Agreement

**FIGURE 2:**
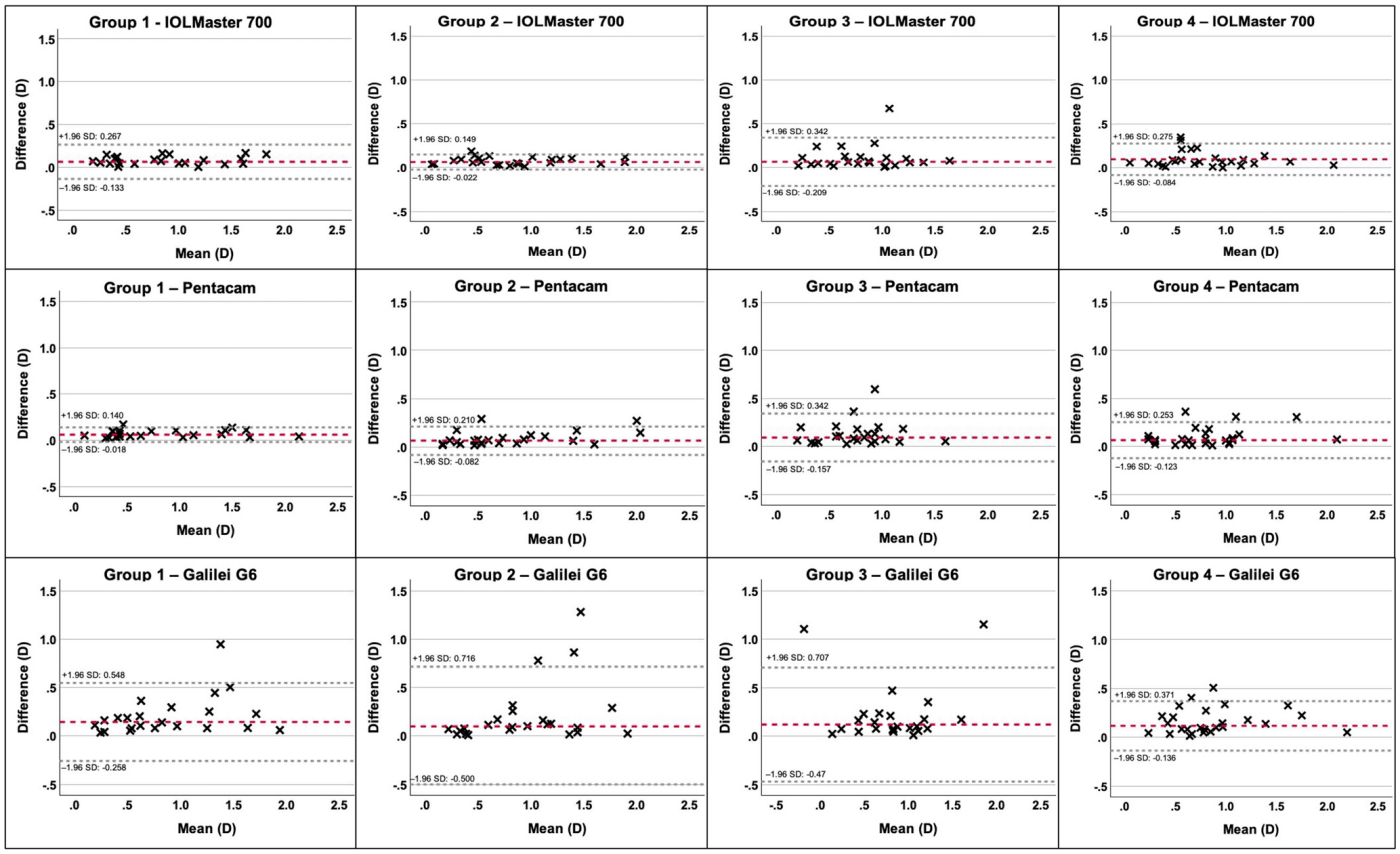
Bland–Altman plots illustrating the mean of the three astigmatism measurements (*x*‐axis) against the average difference in astigmatism values (*y*‐axis) obtained from the three devices, stratified by osmolarity group. D, Dioptres.

The Bland–Altman plots with the limits of agreement of the astigmatism values are shown in Figure 2.

## 4 DISCUSSION

The measurement of corneal tomography and topography and especially preoperative keratometry, which is needed, for example, to correct astigmatism during cataract surgery or refractive lens exchange, relies on precisive measurements with overall good agreement and repeatability of the performing devices. In the present study, the repeatability of keratometry measurements depending on tear film osmolarity with three common biometers was evaluated. In most cases, it showed overall good repeatability of keratometry measurements without a statistically significant influence of tear film osmolarity. Only for the Galilei G6, LOA and ICC values were worse than those of IOLMaster 700 and Pentacam and for vector magnitude analysis, the Galilei G6 showed statistically significant higher differences without influence of tear film osmolarity.

When looking at possible influencing factors, one must consider the different methods of corneal measurements of the three devices. The IOLMaster 700 uses swept‐source optical coherence technology (SS‐OCT) and multidot keratometry and shows good agreement, comparability and repeatability in keratometry and axial length measurements (Kurian et al., 2016; Savini et al., 2021). The Pentacam AXL uses Scheimpflug technology to measure the corneal anterior and posterior curvature with good repeatability and agreement, too (Ho et al., 2008; Shajari et al., 2020). The Galilei G6 combines dual Scheimpflug technology with a Placido disc and performs precise keratometry measurements (Hashemi et al., 2022; McLintock et al., 2025).

The hypothesis of this study was that keratometry measurements tend to be more affected as tear film osmolarity increases. Therefore, devices relying on projection on the corneal surface should be more affected than those relying on OCT‐based methods. The background of this hypothesis is that high osmolarity is associated with higher tear film evaporation which causes a vicious cycle of inflammation and evaporation perpetuating dry eye disease and unstable tear film and thus could affect projection‐based keratometry (Gayton, 2009; Willcox et al., 2017).

A study by Nilsen et al. (2024) showed a greater variability in two repeated measurements with common biometry devices in eyes with signs of dry eye syndrome. Especially when looking at the tear film osmolarity, the study showed a higher variability of astigmatism in eyes with hyperosmolar tear film measurements (>308 mOsm/L or an inter‐eye difference of >9 mOsm/L) for the two devices that use projection‐based keratometry measurements. These findings nearly fit our results where the Galilei G6 showed a higher difference of vector magnitude which is not correlated to a higher tear film osmolarity. Additionally, the IOLMaster 700, which uses a different method of projection‐based keratometry, did not show a higher difference of vector magnitude.

Another study by Epitropoulos et al. found a higher number of eyes in the hyperosmolar group (>316 mOsm/L) with a higher variability of average K measurements and a higher vector difference (Epitropoulos et al., 2015). Here, measurements of both eyes and on different days were evaluated which could explain the higher variability findings.

We detected worse ICC values for the Galilei G6 also shown by the wider spread of the LOA in agreement (Figure 2). Other studies could not confirm those worse ICC values in a bigger patient collective (Lwowski et al., 2025). One must consider possible sample size limitations as the osmolarity groups only consisted of 23–26 patients. Nevertheless, when looking at the overall ICC with the CI for the three devices, the Galilei G6 showed a worse ICC than the other devices in the entire patient population.

Other factors than tear film osmolarity which could possibly affect keratometry measurements must be considered, too. Increasing age has been shown as an associated variable in keratometry measurements (Hashemi et al., 2021). Age distribution between the four groups in our study was not statistically significantly different (Table 1), so age as a contributing factor can be ruled out.

The possible influence of eye drops on topographic measurements is discussed and measurements directly after initial instillation seem to be affected (Lee et al., 2024; Schug et al., 2025), but no influence on possible IOL calculation was found (Jensen et al., 2020). These factors are ruled out, too, as the keratometry measurements were performed before any other diagnostic or therapeutical intervention and the previous application of eye drops was defined as an exclusion criterion.

In comparing the IOLMaster 700 with the Galilei G6, it should be noted that the IOLMaster 700 performs multidot reflection‐based keratometric assessments, while the Galilei G6 determines corneal curvature through the projection of Placido rings onto the corneal surface. Although both techniques are reflection‐based, the IOLMaster 700 captures multiple readings and reports their mean, potentially mitigating measurement fluctuations, especially in eyes with dry eye disease.

Possible limitations of our study are that, due to the manufacturer's recommendation, the objective amount of tear film osmolarity is not utilizable to draw back on the severity of dry eye disease. The division into the different osmolarity groups followed a suggestion of a modified DED severity scale and was conducted in steps of 10 mOsm/L for easier evaluation. Additionally, doubts must be raised that the tear film osmolarity measured in the tear meniscus near to the eyelid fits the osmolarity in the centre of the cornea where all keratometry measurements are performed (Bron et al., 2017; Willcox et al., 2017). The influence of changing humidity and temperature which possibly could affect tear film osmolarity was not evaluated, too, but all measurements were performed randomized in the same room, so possible fluctuations are distributed equally over the four groups. Furthermore, tear film break‐up time may represent a more relevant factor influencing keratometric measurements and therefore warrants further investigation in future studies. In addition, meibomian gland dysfunction should be considered, although current evidence suggests that it does not significantly affect the repeatability of keratometric measurements (Schlatter et al., 2024).

Major strengths of our study include the high patient number calculated by sample size calculation and equal distribution of the patients over the four osmolarity groups. Additionally, three measurements were performed with every device, so repeatability is more clearly emphasized than with performing only two measurements, which a lot of other studies do. Although our osmolarity grading system is not validated, it is supported by other studies that found a positive correlation between DED severity and tear film osmolarity (Lemp et al., 2011; Suzuki et al., 2010; Willcox et al., 2017).

Overall, our results show that, in most cases, tear film osmolarity does not affect keratometry measurements with the three optical biometers. They showed overall good repeatability of keratometry and astigmatism measurements. Unfortunately, the LOA and ICC values for the Galilei G6 showed worse agreement and repeatability than for the IOLMaster 700 and Pentacam, and vector magnitude for the Galilei G6 was statistically significantly different.

## FUNDING INFORMATION

The authors have nothing to report.

## CONFLICT OF INTEREST STATEMENT

KPK: Lecturing for Oculus. TK: Consultant, Research and Lecturing for Alcon, Oculus, Schwind, Staar. Consultant and Lecturing for Tarsus, Ziemer. Research and Lecturing for Teleon Surgical. Consulting for Abbvie, Geuder, LensGen, Santen, Stadapharm, Thieme, Zeiss Meditec. Lecturing for Allergan, Bausch & Lomb, Johnson & Johnson, MedUpdate, streamedup. All other authors have no financial interests to declare.
